# Novel nonsense mutation in gene *CHRNA2* identified by whole-genome sequencing in infant with epilepsy disorder: A case report

**DOI:** 10.1016/j.heliyon.2024.e41484

**Published:** 2024-12-26

**Authors:** Sultan Makhmetov, Kamila Temirkhanova, Saule Rakhimova, Nazerke Satvaldina, Ruslan Kalendar, Ulan Kozhamkulov, Aidos Bolatov, Mirgul Bayanova, Assiya Bazenova, Lyazzat Nazarova, Ainur Akilzhanova, Ulykbek Kairov

**Affiliations:** a Center for Life Sciences, National Laboratory Astana, Nazarbayev University, Kabanbay Batyr Ave 53, Astana, 010000, Kazakhstan; b University Medical Center CF, Kerey-Zhanibek Khandar St. 5/1, 010000, Astana, Kazakhstan

**Keywords:** Epilepsy, Infantile seizures, Acetylcholine receptor alpha 2 subunit, Benign familial infantile seizures (BFIS), Autosomal dominant nocturnal frontal lobe epilepsy (ADNFLE)

## Abstract

Epilepsy is one of the most common neurological disorders affecting approximately 50 million people worldwide. It impacts people of all genders and ages, but evidence suggests a higher incidence rate in children and the elderly.

Given that childhood epilepsy has the risk of causing developmental epileptic encephalopathy, which is associated with intellectual, behavioral, and/or motor disabilities, proper assessment of children with new-onset epilepsy at an early stage is essential to prevent threats affecting neurodevelopmental processes. The aim of this study was to investigate whole genome sequencing data of children diagnosed with epilepsy. Our results revealed an identification of a novel mutation in a 2-year-old male patient who suffered from recurrent epileptic seizures of unknown etiology. The detected variant is heterozygous and located in gene *CHRNA2* (chr8:27321348, NM_000742, c.612G > A, p.Trp204∗) in exon 6. The databases such as Varsome, GeneCards, and NCBI did not reveal any matches with previously identified variants, implying the novelty of the finding. Moreover, according to various prediction tools (MutationTaster, SIFT, CADD, FATHMM-MKL, LRT, DANN, Eigen, and BayesDel), the mutation is characterized as pathogenic, which corresponds to the American College of Medical Genetics and Genomics (ACMG) classification. According to the findings, mutation of the *CHRNA2* gene is closely associated with two disorders known as autosomal dominant nocturnal frontal epilepsy (ADNFLE), and benign familial infantile epilepsy (BFIS). Comparison of proband's clinical manifestations showed that it is difficult to attribute precisely the patient's symptoms to either of the conditions, however the evidence suggests that the patient's symptoms are more consistent with those of ADNFLE. In this report, we expanded the spectrum of existing variations in the *CHRNA2* gene contributing and associated with the development of epilepsy with the important and novel causative genetic variant.

## 1 Introduction

Epilepsy is a chronic brain disorder that is known to affect over 65 million people worldwide with an incidence rate of 50.4–81.7 per 100,000 people per year [1,2]. It is characterized by recurrent, unprovoked seizures accompanied by an excessive discharge of electrical activity within the brain that temporarily alters behavior [3]. To date, more than 20 types of epilepsy have been documented, the causes of which seem to be rather complex and highly heterogeneous [4]. People of all genders were shown to be affected equally, but some differences in regard to age were also observed. As evidenced by the bimodal distribution of incidence in different age groups, young children and the elderly are at the greatest risk of developing the disease [5,6].

Recent advancements in gene sequencing technologies, computational methods, and collaborative efforts have improved our understanding of the genetic basis of the disorder. It is now widely accepted that genetics play a more significant role in epilepsy than previously believed. Many forms of epilepsy result from genetic mutations that cause synaptic, receptor, and ion concentration abnormalities, which affect membrane excitability [7]. With the increasing sophistication and accessibility of genetic testing, more of these genetic alterations are being discovered.

This report presents the case of a 2-year-old boy with epileptic seizures, who has a new heterozygous nonsense mutation in *CHRNA2* (exon 6). Previously identified variants have been found in the same genomic region as in our case, in exon 6, however, most of them are missense mutations causing alterations in chemical interaction, as well as ligand binding affinity of the receptor. Regarding the identified mutation, it is important to emphasize its unique nature, acquisition of a premature stop codon, which is hypothesized to lead to a significant reduction in the *CHRNA2* protein.

Here we present the novel and validated genetic variant and discuss a potentially important and significant role as the genetic biomarker in the development of early-onset epilepsy.

## 2 Case presentation

The proband, a 2-year-old boy, is one of the three siblings from a non-consanguineous marriage, who presented various episodes of seizures since the beginning of life. He was initially referred to the National Maternal and Child Research Center, however, due to multiple episodes of seizures of unknown origin refractory to antiepileptic therapy he came to our attention (Supplementary Table 2. Timeline).

**Fig. 1:**
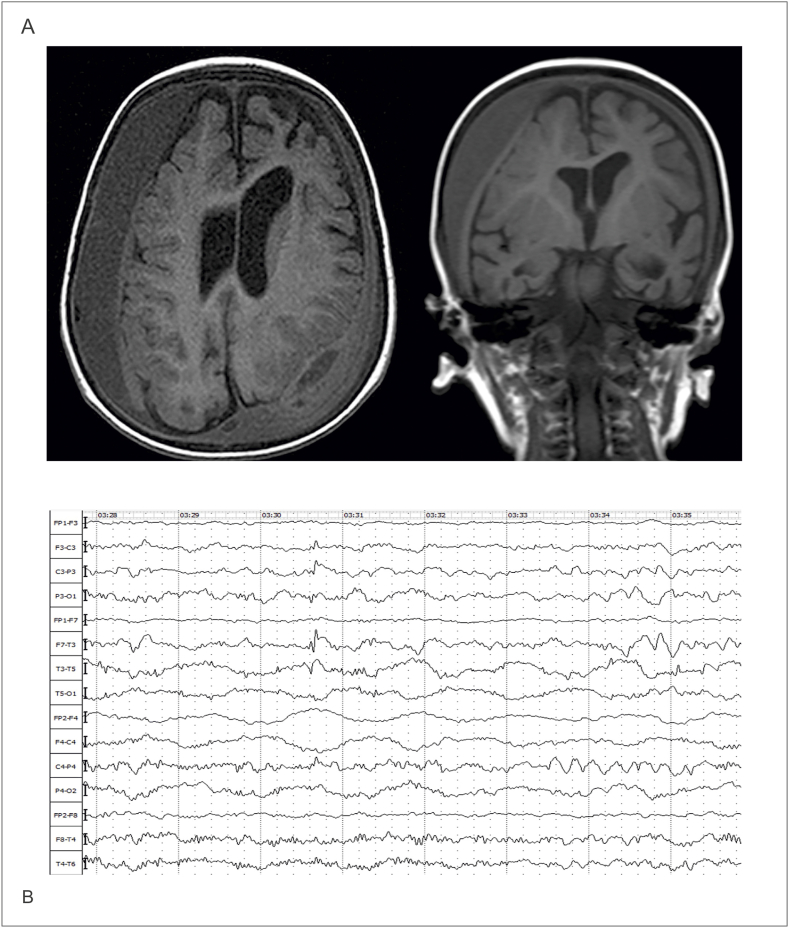
**Diagnostic results of the proband. a)** The Magnetic Resonance Imaging (MRI) of the proband revealed signs of cerebral hemisphere sub-atrophy. Bilateral subdural hygromas were present, with the right side being more pronounced (18 mm) compared to the left (5 mm). A similar encysted liquid formation, in the shape of a biconvex lens, was observed in the parietal-occipital region on the left and measured 1.2 × 2.6 cm in size with clear, even contours. Additionally, a small retro cerebellar cyst was present, causing the retro cerebellar space to expand to 22 × 9 mm in the sagittal projection. ***b)****The proband's EEG revealed disrupted bioelectrical activity in the wakefulness-sleep cycle. During wakefulness, diffuse beta-wave activity was observed throughout the recording, with no clear determination of the basic rhythm. Epileptiform activity was recorded in the form of acute-slow wave complexes in the left central leads that spread to the parietal and temporal leads, and sometimes showed asynchronous bilateral spread. During sleep, diffuse theta/alpha-like waves were observed, with a maximum in the fronto-central leads and accompanied by diffuse beta-wave activity, resulting in a fragmented sleep structure. Low-index sleep spindles were recorded and in dreams, sharp-slow wave complexes were detected in the central and parietal leads. Additionally, flashes of diffuse delta waves with maximum amplitude in the central and temporal leads were recorded during sleep with a high index. The EEG trace was also found to have artifacts related to motor activity*.

The first onset of the disorder occurred on the second day of proband's life. He was in intensive care for the period of one month with an initial diagnosis of neonatal convulsions. Phenobarbital (5 mg/kg/day) was prescribed as a first-line treatment but was ineffective. Depakine was then introduced, with the gradually increased dosage from 15 mg/day to 200 mg/day. When this proved ineffective, Levetiracetam (150 mg/day) was added. Despite these adjustments, seizures and myoclonic spasms persisted, leading to the replacement of Depakine with Sodium valproate (150 mg/day), which stopped convulsions but caused frequent vomiting. Therefore, due to the overall ineffectiveness of the intervention, the exact diagnosis remained in doubt. Evaluation using magnetic resonance imaging (MRI) indicated severe perinatal lesions of the CNS of hypoxic-ischemic origin, also signs of sub-atrophy of the cerebral hemispheres (Fig. 1a). Electroencephalography (EEG) showed disrupted bioelectrical activity with diffuse beta waves, left central epileptiform discharges, and fragmented sleep with reduced spindles and sharp-slow wave complexes, supporting severe epileptic encephalopathy (Fig. 1b). Until 4 months, the patient had several episodes of tonic-clonic convulsions of the upper and lower extremities in the form of periodic rhythmic twitches: as well as myoclonic spasms. By the age of 1 year, the patient had experienced an exacerbation of seizures, manifested in the form of myoclonus and fading lasting more than 5 minutes, which further intensified and increased to 30–40 minutes. Antiepileptic treatment was changed several times depending on the side effects of the drugs and the overall outcome. However, due to the inadequacy of the results of treatment, a diagnosis of epileptic encephalopathy with polymorphic seizures, a resistant form, was made. After discharge, the patient's condition improved. The previous seizures stopped, but the mother noted the appearance of involuntary movements and developmental delay. It was noted that the child had poor sleep, disturbing awakenings, and nightmares in the form of episodes during sleep/night, as well as sudden movements of the upper limbs during sleep. At the age of 2 years old, seizures reappeared, and new symptoms included twitching of mimic and chewing muscles.

According to the parents, a family history review did not reveal any relatives with epilepsy, except proband. His older brother and older sister had no signs of a similar disease, also father and mother were phenotypically normal.

## 3 Molecular/genetic analysis

A peripheral blood sample was taken from the proband (II:3), and whole genome sequencing was done to identify a possible mutations causing epilepsy. A total of 150 top-ranked genes associated with epilepsy were chosen from a GeneCards database (Supplementary Table 3. List of Genes). Filtering for highly associated genes with epilepsy resulted in 51 non-synonymous variants and 1 stop gain variant (Supplementary Table 4. Identified Genetic Variants). Among them, four novel mutations were identified as pathogenic by various predictor tools (Varsome and MutationTaster [8]). These included three nonsynonymous mutations: *PLPBP*: c.T20A (p.Met7Lys), *PLPBP*: c.A487G (p.Lys163Glu), *CPA6*: c.G973T (p.Ala325Ser), and one stop-gain mutation: *CHRNA2:* c.612G > A (p.Trp204∗).

**Fig. 2:**
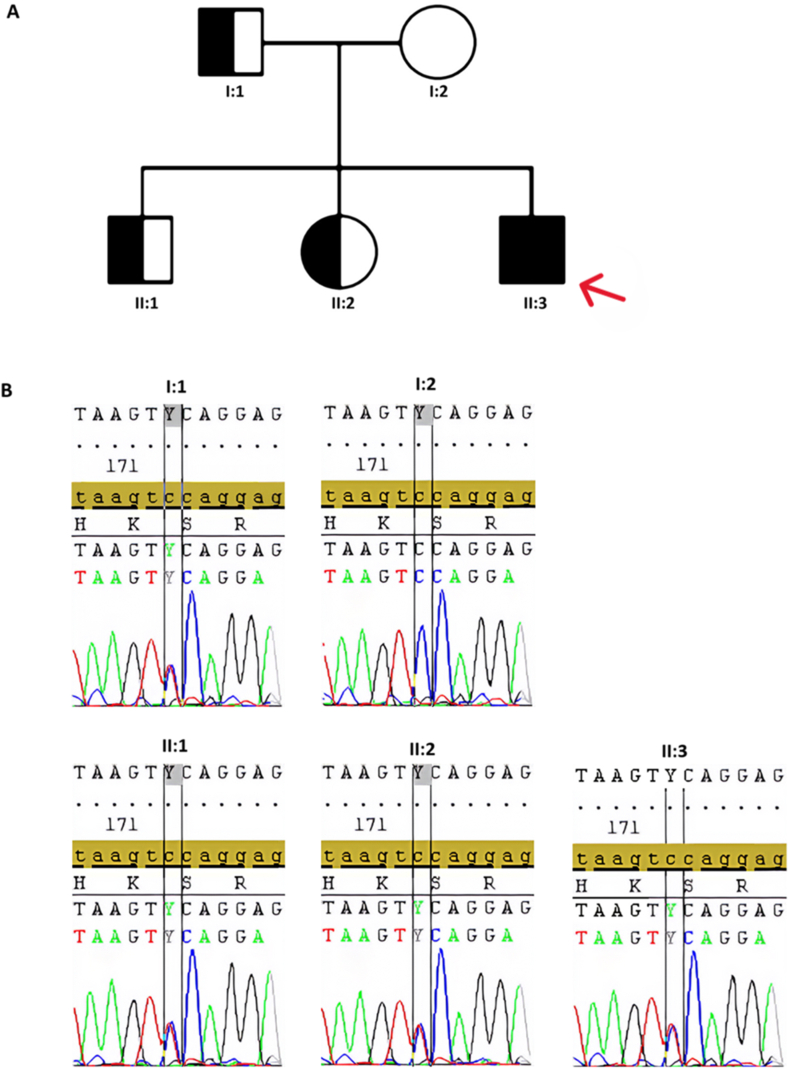
**Family description. a)** The pedigree chart illustrates the family history of the proband. In this chart, squares represent males, and circles represent females. Open symbols indicate healthy individuals who do not carry the mutation (I-2), while partially filled symbols represent individuals who are carriers of the heterozygous CHRNA2 mutation (I-1, II-1, II-2). Fully filled symbols denote individuals affected by the phenotype (II-3). The proband (II-3) is identified as the affected individual, whereas the father (I-1), older brother (II-1), and older sister (II-2) are categorized as healthy carriers of the mutation. **b)** Sanger sequencing results of the family.

However, since all the identified nonsynonymous mutation variants were heterozygous and the associated disorders followed an autosomal recessive inheritance pattern, we considered that their effect on the patients' phenotype is likely to be minor. Consequently, because mutations in the *CHRNA2* gene primarily exhibit an autosomal dominant inheritance pattern, we concentrated on the novel stop-gain variant c.612G > A (p.Trp204∗). This variant has not been previously reported in databases such as Varsome, GeneCards, ClinVar, or the 1000 Genomes Project [9]. Located in the 6th exon (out of 7) of the *CHRNA2* gene, this heterozygous variant was initially classified as “pathogenic” by the Varsome platform. This classification was further validated by several predictive tools, including SIFT, CADD, MutationTaster, DANN, LRT, FATHMM-MKL, and Eigen (see Supplementary Table 5: Predictor Tools Summary). In accordance with the American College of Medical Genetics (ACMG) guidelines, this variant is classified as "pathogenic" based on a combination of criteria, including strong, moderate, and supporting evidence of its pathogenicity (PSV1, PM2, PP3 respectively) (Supplementary Table 6. ACMG Classification of the variant) [10]. The c.612G > A (p.Trp204∗) in the *CHRNA2* variant was validated using Sanger sequencing (Fig. 2a and b) and confirmed the presence of a heterozygous mutation in the proband (II-3). *Because the mutations in CHRNA2 follow an autosomal dominant mode of inheritance,* we expected that only proband had a mutation in the family, since all family members except proband were clinically healthy. However, according to Sanger sequencing the proband's father (I-1), older brother (II-1), and sister (II-2) also carried the same heterozygous variant, but the mother (I-2) did not. The obtained results showed that the patient inherited the variant from his father.

## 4 Discussion/conclusion

In the current study, we described a child with a new heterozygous mutation in the *CHRNA2* gene: c.612G > A (p.Trp204∗), which leads to the acquisition of a premature stop codon. The variant was suggested to be pathogenic; assessment of the mutation site with a range of predictor tools and ACMG-based interpretation criteria further supported our conjectures. In overall we performed seven different predictions using DANN, CADD, SIFT, LRT, MutationTaster, Eigen, and FATHMM-MKL to determine the possible effect of the amino acid substitution. The p.Trp204∗ has been classified as pathogenic by all the tools listed. Perhaps this is due to the fact that the point mutation at position 204 observed in our case leads to early termination of translation, i.e., to the loss of the remaining 326 amino acids that make up more than half of the protein (Supplementary Table 7. Comparison of sequences). Such radical changes can potentially result in the expression of truncated and non-functional protein products, which are then degraded in the cell due to low stability**.**

The *CHRNA2* gene encodes the alpha-2 subunit of the nicotinic acetylcholine receptors, ligand-gated pentameric ion channels consisting of sequences of alpha and beta subunits [11]. Up to now 12 nAChR subunits have been identified (α2–α10 and β2–β4). Each of them consists of 4 transmembrane domains(M1-M4) and in different combinations give rise to unique receptor subtypes [12]. These receptors are expressed in the peripheral and central nervous systems and are involved in rapid neurotransmission [13]. According to postmortem examination in humans, α2 subunit exhibits its highest expression within thalamus, whereas expression in hippocampus and subcortical nuclei is moderate to weak [14]. However, the existing uncertainties regarding the function of the α2 subunits do not allow us to clearly state the detrimental effect of the identified variant. The pathogenic bioinformatics predictions, clinical manifestations of the patient, and results of previous studies allow us to only make conjectures about the effects of the mutations.

Variations in *CHRNA2* have previously been shown to play a causal role in autosomal dominant nocturnal frontal lobe epilepsy (Supplementary Table 8. Literature Review of *CHRNA2* Gene Mutations). As identified by Aridon et al. [14], a heterozygous single nucleotide substitution of hydrophobic isoleucine for polar asparagine in the *CHRNA2* gene (exon 6) induces epileptogenesis associated with sleep and awakening mechanisms, establishing link with ADNFLE. It has been suggested that this mutation affects the transition between closed and open states of nAchR, which, as a consequence, leads to an increase in the sensitivity of the mutant receptor to lower cholinergic stimuli and, presumably, to the stimulation of seizures by facilitating synchronous spontaneous oscillations on the thalamic and cortical levels. Confirming the results, Conti and colleagues conducted another study in which the Ile297Phe mutation of the same gene was tested in vitro to determine its functional effect [15]. The mutation is localized in the first amino acid of M2 (transmembrane domain 2) and, conversely, to the previous mutation, has negligible effect on the receptor sensitivity to nicotine, but causes a complete loss of expression in homozygosity, and partial loss in heterozygosity pointing to a loss-of-function mechanism. This aligns with the loss-of-function character of the p.Trp204∗ mutation reported in our study, suggesting that such mechanisms may be common among epilepsy-associated *CHRNA2* mutations, though some exceptions exist. According to the authors, such drastic changes are due to the involvement of the affected amino acid in the formation of channel pore, thus passage of the current. Another publication in the ClinVar database highlights the potential association of another *CHRNA2* mutation (Arg376Trp) with benign familial infantile seizures (BFIS), but this has yet to be confirmed [16]. The authors suggest that the c.1126c > T:p.Arg376Trp mutation alters the chemical interactions within the *CHRNA2* gene, potentially explaining its disease-causing impact. The highly conserved arginine residue is situated near the M1 and M4 transmembrane helices, where it participates in hydrogen bonds that aid in stabilizing the α-helix structure. The residue also forms salt bonds with the highly conserved asp484. Therefore, the conversion of arginine 376 to tryptophan disrupts these interactions, which may be the underlying cause of its harmful influence. These results underscore the crucial role of the *CHRNA2* gene in multiple forms of epilepsy and suggest that mutations in this gene can have a significant adverse effect on health by affecting its function.

Since these works describe mutations that are close to our case, we have chosen common characteristics of ADNFLE and BFIS (Supplementary Table 9. Comparison of clinical manifestations of BFIS, ADNFLE, and proband) to compare them with the symptoms observed in our proband. What has been found is that BFIS usually resolves after the first year of life and does not affect neurodevelopment, but this is not consistent with what is observed in our patient who still has seizures at 2 years of age. ADNFLE, on the other hand, is a lifelong condition that can result in developmental delays [17]. Our patient has been noted to have a psychomotor arrest, which is coherent with the lifelong impact of ADNFLE. Moreover, the patient's symptoms of poor sleep, fearful awakenings, nightmares, and nocturnal seizures correspond to characteristics of ADNFLE. In contrast, as indicated by parents, the family of the proband had no history of epilepsy. At the same time, the father, older brother, and older sister carried the same heterozygous mutation such as proband. This phenomenon of variable penetrance is unusual for both BFIS and ADNFLE, which usually exhibit high penetrance in their autosomal dominant inheritance pattern. The cause for such low penetrance in this case remains unclear and requires further and functional investigations on model organisms. Overall, while it is difficult to definitively attribute the patient's symptoms to either ADNFLE or BFIS, the evidence suggests that the patient's symptoms are more consistent with those of ADNFLE.

In conclusion, we found a novel heterozygous variant in *CHRNA2* gene c.612G > A (p.Trp204∗), which leads to the acquisition of a premature stop codon and associated with epileptogenesis. This variant is expanded spectrum of mutations in *CHRNA2* gene and according to clinical symptoms related to autosomal dominant nocturnal frontal epilepsy. Considering the existence of an unaffected carrier in the family, ADNFLE is possible to be early diagnosed clinically. Genetic variant identification providing support to conclusive diagnosis in clinical practice and essential for sporadic cases with unknown etiology, as well as for their members.

## CRediT authorship contribution statement

**Sultan Makhmetov:** Writing – original draft, Visualization, Software, Investigation, Formal analysis. **Kamila Temirkhanova:** Writing – original draft, Visualization, Validation, Software, Investigation, Formal analysis. **Saule Rakhimova:** Validation, Methodology, Investigation. **Nazerke Satvaldina:** Validation, Methodology. **Ruslan Kalendar:** Methodology, Formal analysis. **Ulan Kozhamkulov:** Methodology. **Aidos Bolatov:** Resources, Project administration, Funding acquisition, Data curation, Conceptualization. **Mirgul Bayanova:** Resources, Methodology, Data curation. **Assiya Bazenova:** Resources, Formal analysis, Data curation. **Lyazzat Nazarova:** Resources, Methodology, Data curation. **Ainur Akilzhanova:** Writing – review & editing, Methodology, Funding acquisition, Formal analysis, Conceptualization. **Ulykbek Kairov:** Writing – review & editing, Writing – original draft, Visualization, Supervision, Software, Methodology, Investigation, Funding acquisition, Formal analysis, Conceptualization.

## Ethics statement

The study was approved by the local commission on bioethics of the University Medical Center CF, an extract from Protocol No.1 dated June 29, 2021. Written informed consent and permission to publish data were obtained from parents for all research investigations.

## Data availability

All data available by request to corresponding author. Sequencing data have been deposited at National Center for Biotechnology Information Sequence Read Archive under accession number PRJNA956710 (https://www.ncbi.nlm.nih.gov/bioproject/PRJNA956710).

## Funding

This study was funded by the Science Committee of the Ministry of Science and Higher Education of the Republic of Kazakhstan (Grants No. AP23490594, BR18574184, BR24993023, BR24992841, BR27199879), 10.13039/501100012632Nazarbayev University funding CRP grants 021220CRP2222, 211123CRP1608 and carried out as part of a joint pilot project between the “UMC” CF and the “NLA” PI (contract DPMRV-1986/161-20)

## Declaration of competing interest

The authors declare that they have no known competing financial interests or personal relationships that could have appeared to influence the work reported in this paper.
