# Supplementary material for: Novel nonsense mutation in gene CHRNA2 identified by whole-genome sequencing in infant with epilepsy disorder: A case report
Source: Heliyon. 2024 Dec 26;11(1):e41484. doi: 10.1016/j.heliyon.2024.e41484 (PMC11743308; doi:10.1016/j.heliyon.2024.e41484)
Supplement: Multimedia component 1 [file mmc1.docx]

­

**Table 1**. Oligonucleotide primers used for genomic sequencing analysis.

| **ID** | **Primer sequence**  **(5'-3')** | **Tm (°C)*** | **GC (%)** | **LC (%)**** | **Location** | **GenBank accession** | **PCR product (bp)** |
| --- | --- | --- | --- | --- | --- | --- | --- |
| adra2bF | ctgtggtcattggcgttt | 58.7 | 50.0 | 81 | 6121→6138 | NG_032950 | 357 |
| adra2bR | atgaggcctacaggatct | 57.2 | 50.0 | 86 | 6460←6477 |  |  |
| 36umcF | aatgcacagcgtgatctt | 57.5 | 44.4 | 92 | 20316→20333 | NG_015827 | 442 |
| 36umcR | gtttgcagtgacccacat | 58.4 | 50.0 | 83 | 20740←20757 |  |  |

* Melting temperature (Tm) calculated for oligonucleotide concentration of 200 nM in 55 mM KCl with 1 mM Mg^2+^;

** Linguistic sequence complexity (LC) (%);

PCR reactions were carried out in a 25 µL reaction mixture, containing 25 ng of template DNA, 1× Phusion HF Buffer with 1.5 mM MgCl_2_, 0.2 µM of each primer, 0.25 mM of each dNTP, and 1 U of Phusion Hot Start II DNA Polymerase (Thermo Fisher Scientific Inc., USA). Amplification was performed using a SimpliAmp™ Thermal Cycler (Thermo Fisher Scientific Inc., USA) with the following conditions: an initial denaturation step at 98 °C for 1 min, followed by 32 amplification cycles at 98 °C for 5 s, 60 °C for 30 s, and 72 °C for 10 s, and a final extension at 72 °C for 1 min.

**1** Kalendar R, Khassenov B, Ramankulov Y, Samuilova O, Ivanov KI 2017. FastPCR: an *in silico* tool for fast primer and probe design and advanced sequence analysis. Genomics, 109(4-5): 312-319. DOI:10.1016/j.ygeno.2017.05.005

**2** Kalendar R, Lee D, Schulman AH 2011. Java web tools for PCR, *in silico* PCR, and oligonucleotide assembly and analysis. Genomics, 98(2): 137-144. DOI:10.1016/j.ygeno.2011.04.009
