# Supplementary material for: Novel nonsense mutation in gene CHRNA2 identified by whole-genome sequencing in infant with epilepsy disorder: A case report
Source: Heliyon. 2024 Dec 26;11(1):e41484. doi: 10.1016/j.heliyon.2024.e41484 (PMC11743308; doi:10.1016/j.heliyon.2024.e41484)
Supplement: Multimedia component 2 [file mmc2.docx]

**Supplementary Table 1. Timeline**

| **Date** | **Event** | **Details** |
| --- | --- | --- |
| **17.09.2020**  **Second day of life** | **Clonic convulsions lasting several seconds** | **Stopped by Diazepam** |
| **~ 1 month in ICU** | **Diagnosis of neonatal convulsions** | **Phenobarbital (5mg/kg/day) prescribed** |
| **1.5 months** | **Tonic-clonic seizures observed** | **Phenobarbital stopped, replaced by Depakine syrup (15 mg/day)** |
|  |  | **Dosage increased to 200 mg/day**  **Depakine showed no effect, Levetiracetam (150 mg/day) added**  **Clonic seizures increased, myoclonic spasms appeared**  **Depakine replaced by Sodium valproate (150 mg/day)**  **Convulsions stopped, but child began vomiting frequently** |
|  |  |  |
|  |  |  |
|  |  |  |
|  |  |  |
| **4 months** | **Sodium valproate changed to Depakine (150 mg/day)** | **Levetiracetam continued (100 mg/day)** |
|  |  | **Tonic-clonic convulsions and myoclonic spasms reappeared, vomiting stopped**  **Diagnosis: focal epilepsy** |
|  |  |  |
| **11.03.2021** | **EEG** |  |
| **15.03.2021** | **MRI** |  |
| **24.09.2021**  **1 year, 7 days** |  | **Increase in body temperature without catarrhal manifestations** |
| **26.09.2021**  **1 year, 9 days** |  | **Seizures transitioned to myoclonus and fading, lasted more than 5 minutes** |
|  |  | **Treated with Diazepam, prednisolone, and Analgin-Dimedrol, admitted to children's hospital** |
| **15.10.2021** | **Department of Pediatric Neurology** | **Status epilepticus developed** |
| **12.11.2021** | **Right-sided pneumonia with pneumofibrosis** | **Child underwent tracheostomy** |
| **16.11.2021-20.11.2021** | **Department of Neurology** | **Episodes of generalized and clonic seizures intensified, lasting up to 30-40 minutes** |
| **30.11.2021** | **MRI** |  |
| **06.12.2021** | **Increase in frequency of seizures, electrolyte imbalance, vomiting, hypoalbuminemia, edema** | **Antiepileptic drugs repeatedly changed** |
| **26.12.2021** | **Diagnosis: Epileptic encephalopathy with polymorphic seizures, resistant form** |  |
| **After discharge** | **Improved condition, previous seizures stopped** | **Involuntary movements and developmental delay observed** |
| **21.02.2022** | **EEG** |  |
| **24.02.2022** | **MRI** |  |
| **18.08.2022-25.08.2022** | **Inpatient treatment** | **Improvement, seizures stopped** |
| **19.09.2022** | **Seizures repeated in form of twitching and chewing muscles** | **Poor sleep, fearful awakenings, nightmares, and sudden limb movements observed** |
| **27.10.2022-02.11.2022** | **Admission for treatment adjustment** |  |
| **28.10.2022** | **Angiopathy of the retina** | **Damage to visual analyzer above 3 neurons not ruled out** |
| **31.10.2022** | **Ultrasound: Hepatomegaly** |  |
