# Supplementary material for: Novel nonsense mutation in gene CHRNA2 identified by whole-genome sequencing in infant with epilepsy disorder: A case report
Source: Heliyon. 2024 Dec 26;11(1):e41484. doi: 10.1016/j.heliyon.2024.e41484 (PMC11743308; doi:10.1016/j.heliyon.2024.e41484)
Supplement: Multimedia component 6 [file mmc6.docx]

**Supplementary Table 6.** ACMG Classification of the variant [1]

| **1. Very strong evidence of pathogenicity**   - **PSV1** (present) **-** Null variant (nonsense, frameshift, canonical +/−1 or 2 splice sites, initiation codon, single or multi-exon deletion) in a gene where loss of function (LOF) is a known mechanism of disease. | **Present**  *(CHRNA2 variant is nonsense mutation)* |
| --- | --- |
| **2. Strong evidence of pathogenicity**   - **PS1, PS2, PS3, PS4** | **Not present** |
| **3. Moderate evidence of pathogenicity**   - **PM2 -** Absent from controls (or at extremely low frequency if recessive) in Exome Sequencing Project, 1000 Genomes or ExAC | **Present** |
| - **PM1, PM3, PM4, PM5, PM6** | **Not present** |
| **4. Supporting evidence of pathogenicity**   - **PP3** - Multiple lines of computational evidence support a deleterious effect on the gene or gene product (conservation, evolutionary, splicing impact, etc) | **Present** |
| - **PP1, PP2, PP4, PP5** (not present) | **Not present** |

**Rules for Combining Criteria to Classify Sequence Variant**

- 1 Very Strong (PSV1) + 1 Moderate (PM1-PM6) and 1 Supporting (PP1-PP5) classify the variant as pathogenic.

**References:**

1 - Richards, S., Aziz, N., Bale, S., Bick, D., Das, S., Gastier-Foster, J., Grody, W. W., Hegde, M., Lyon, E., Spector, E., Voelkerding, K., Rehm, H. L., & ACMG Laboratory Quality Assurance Committee (2015). Standards and guidelines for the interpretation of sequence variants: a joint consensus recommendation of the American College of Medical Genetics and Genomics and the Association for Molecular Pathology. *Genetics in medicine : official journal of the American College of Medical Genetics*, *17*(5), 405–424. <https://doi.org/10.1038/gim.2015.30>
