# Supplementary material for: Novel nonsense mutation in gene CHRNA2 identified by whole-genome sequencing in infant with epilepsy disorder: A case report
Source: Heliyon. 2024 Dec 26;11(1):e41484. doi: 10.1016/j.heliyon.2024.e41484 (PMC11743308; doi:10.1016/j.heliyon.2024.e41484)
Supplement: Multimedia component 7 [file mmc7.docx]

**Comparison of aa sequences (wt vs. mutated)**

| **Wildtype AA sequence:**  **Position (AA) of stopcodon in wt:** 530 | MGPSCPVFLS FTKLSLWWLL LTPAGGEEAK RPPPRAPGDP LSSPSPTALP QGGSHTETED RLFKHLFRGY NRWARPVPNT SDVVIVRFGL SIAQLIDVDE KNQMMTTNVW LKQEWSDYKL RWNPTDFGNI TSLRVPSEMI WIPDIVLYNN ADGEFAVTHM TKAHLFSTGT VHWVPPAIYK SSCSIDVTFF PFDQQNCKMK FGSWTYDKAK IDLEQMEQTV DLKDYWESGE WAIVNATGTY NSKKYDCCAE IYPDVTYAFV IRRLPLFYTI NLIIPCLLIS CLTVLVFYLP SDCGEKITLC  ISVLLSLTVF LLLITEIIPS TSLVIPLIGE YLLFTMIFVT LSIVITVFVL NVHHRSPSTH TMPHWVRGAL LGCVPRWLLM NRPPPPVELC HPLRLKLSPS YHWLESNVDA EEREVVVEEE DRWACAGHVA PSVGTLCSHG HLHSGASGPK AEALLQEGEL LLSPHMQKAL EGVHYIADHL RSEDADSSVK EDWKYVAMVI DRIFLWLFII VCFLGTIGLF LPPFLAGMI* |
| --- | --- |
| **Mutated AA sequence:** c.612G>A(p.Trp204Ter), W204X  **Position (AA) of stopcodon in wt / mu AA sequence:** 530 / 204 | MGPSCPVFLS FTKLSLWWLL LTPAGGEEAK RPPPRAPGDP LSSPSPTALP QGGSHTETED RLFKHLFRGY NRWARPVPNT SDVVIVRFGL SIAQLIDVDE KNQMMTTNVW LKQEWSDYKL RWNPTDFGNI TSLRVPSEMI WIPDIVLYNN ADGEFAVTHM TKAHLFSTGT VHWVPPAIYK SSCSIDVTFF PFDQQNCKMK  FG**S*** |
| **Mutated AA sequence:** c.1126C>T  (p.Arg376Trp), R376W  **Position (AA) of stopcodon in wt / mu AA sequence:** 530 / 530 | MGPSCPVFLS FTKLSLWWLL LTPAGGEEAK RPPPRAPGDP LSSPSPTALP QGGSHTETED RLFKHLFRGY NRWARPVPNT SDVVIVRFGL SIAQLIDVDE KNQMMTTNVW LKQEWSDYKL RWNPTDFGNI TSLRVPSEMI WIPDIVLYNN ADGEFAVTHM TKAHLFSTGT VHWVPPAIYK SSCSIDVTFF PFDQQNCKMK FGSWTYDKAK IDLEQMEQTV DLKDYWESGE WAIVNATGTY NSKKYDCCAE IYPDVTYAFV IRRLPLFYTI NLIIPCLLIS CLTVLVFYLP SDCGEKITLC  ISVLLSLTVF LLLITEIIPS TSLVIPLIGE YLLFTMIFVT LSIVITVFVL NVHHRSPSTH TMPHWVRGAL LGCV**PW**WLLM NRPPPPVELC HPLRLKLSPS YHWLESNVDA EEREVVVEEE DRWACAGHVA PSVGTLCSHG HLHSGASGPK AEALLQEGEL LLSPHMQKAL EGVHYIADHL RSEDADSSVK EDWKYVAMVI DRIFLWLFII VCFLGTIGLF LPPFLAGMI* |
| **Mutated AA sequence:** c.889A>T (p.Ile297Phe), I297F  **Position (AA) of stopcodon in wt / mu AA sequence:** 530 / 530 | MGPSCPVFLS FTKLSLWWLL LTPAGGEEAK RPPPRAPGDP LSSPSPTALP QGGSHTETED RLFKHLFRGY NRWARPVPNT SDVVIVRFGL SIAQLIDVDE KNQMMTTNVW LKQEWSDYKL RWNPTDFGNI TSLRVPSEMI WIPDIVLYNN ADGEFAVTHM TKAHLFSTGT VHWVPPAIYK SSCSIDVTFF PFDQQNCKMK FGSWTYDKAK IDLEQMEQTV DLKDYWESGE WAIVNATGTY NSKKYDCCAE IYPDVTYAFV IRRLPLFYTI NLIIPCLLIS CLTVLVFYLP SDCGE**KF**TLC  ISVLLSLTVF LLLITEIIPS TSLVIPLIGE YLLFTMIFVT LSIVITVFVL NVHHRSPSTH TMPHWVRGAL LGCVPRWLLM NRPPPPVELC HPLRLKLSPS YHWLESNVDA EEREVVVEEE DRWACAGHVA PSVGTLCSHG HLHSGASGPK AEALLQEGEL LLSPHMQKAL EGVHYIADHL RSEDADSSVK EDWKYVAMVI DRIFLWLFII VCFLGTIGLF LPPFLAGMI* |
| **Mutated AA sequence:** c.836T>A (p.Ile279Asn), I279N  **Position (AA) of stopcodon in wt / mu AA sequence:** 530 / 530 | MGPSCPVFLS FTKLSLWWLL LTPAGGEEAK RPPPRAPGDP LSSPSPTALP QGGSHTETED RLFKHLFRGY NRWARPVPNT SDVVIVRFGL SIAQLIDVDE KNQMMTTNVW LKQEWSDYKL RWNPTDFGNI TSLRVPSEMI WIPDIVLYNN ADGEFAVTHM TKAHLFSTGT VHWVPPAIYK SSCSIDVTFF PFDQQNCKMK FGSWTYDKAK IDLEQMEQTV DLKDYWESGE WAIVNATGTY NSKKYDCCAE IYPDVTYAFV IRRLPLFYTI NLIIPCL**LN**S CLTVLVFYLP SDCGEKITLC  ISVLLSLTVF LLLITEIIPS TSLVIPLIGE YLLFTMIFVT LSIVITVFVL NVHHRSPSTH TMPHWVRGAL LGCVPRWLLM NRPPPPVELC HPLRLKLSPS YHWLESNVDA EEREVVVEEE DRWACAGHVA PSVGTLCSHG HLHSGASGPK AEALLQEGEL LLSPHMQKAL EGVHYIADHL RSEDADSSVK EDWKYVAMVI DRIFLWLFII VCFLGTIGLF LPPFLAGMI* |
