# Supplementary material for: Novel nonsense mutation in gene CHRNA2 identified by whole-genome sequencing in infant with epilepsy disorder: A case report
Source: Heliyon. 2024 Dec 26;11(1):e41484. doi: 10.1016/j.heliyon.2024.e41484 (PMC11743308; doi:10.1016/j.heliyon.2024.e41484)
Supplement: Multimedia component 8 [file mmc8.docx]

**Supplementary Table 5. BFIS and ADNFLE**

| **Author and year of publication** | **ClinVar Database** | **Mutation type** | **Position** | **Outcome** | **Associated disorder** |
| --- | --- | --- | --- | --- | --- |
| *Trivisano M*, 2015  <https://onlinelibrary.wiley.com/doi/full/10.1111/epi.12967> | Uncertain significance | Heterozygous missense mutation | c.1126 C>T; p. Arg376Trp)  Located in the cytoplasmic domain of receptor subunit  close to the M3 transmembrane domain | Impaired chemical interactions | Benign familial infantile seizures (BFIS). |
| *Conti V*, 2015  <https://pubmed.ncbi.nlm.nih.gov/25770198/> | Pathogenic | Missense mutation | c.889A>T (p.Ile297Phe)  First amino acid of the second transmembrane domain of CHRNA2 | Complete loss of expression in homozygosity and a decrease to about 40% in heterozygosity, resulting in the loss of receptor function | ADNFLE |
| Aridon P, 2006  <https://www.ncbi.nlm.nih.gov/pmc/articles/PMC1559502/> | Pathogenic | Heterozygousmissense mutation | 8p12.3-8q12.3  836T→A in exon 6  I279N, in the first transmembrane domain of CHRNA2 subunit | Increase in the receptor sensitivity to acetylcholine | ADNFLE |
| *Dash B*, 2014  <https://www.ncbi.nlm.nih.gov/pmc/articles/PMC4135378/> |  | Missense mutation | rs141072985: D478N and rs563447740: D478E  Predicted to be part of  cytoplasmic amphipathic α-helices that typically precedes the TM IV of a typical nAChR subunit | D478E variation increases the current responses (Imax) of α2β2- and α2β4-nAChRs  D478N variation only increases the Imax of α2β2 nAChRs |  |
| *Chiara V*, 2019  <https://www.frontiersin.org/articles/10.3389/fnmol.2019.00017/full> |  | Missense mutation | T>C transition at cDNA position 754 (c.754T>C), p.Tyr252His CHRNA2 mutation located in the N-terminal ligand-binding domain | Decrease of the affinity of the ligand binding site for the agonist ACh, resulting in loss of function mutation | ADNFLE |
