# Supplementary material for: Novel nonsense mutation in gene CHRNA2 identified by whole-genome sequencing in infant with epilepsy disorder: A case report
Source: Heliyon. 2024 Dec 26;11(1):e41484. doi: 10.1016/j.heliyon.2024.e41484 (PMC11743308; doi:10.1016/j.heliyon.2024.e41484)
Supplement: Multimedia component 9 [file mmc9.docx]

| **Supplementary Table 6.** Comparison of clinical manifestations of BFIS, ADNFLE, and proband | | |
| --- | --- | --- |
| **Benign Familial Infantile Seizure**  **(BFIS)** | **Autosomal Dominant Nocturnal Frontal Lobe Epilepsy (ADNFLE)** | **Proband** |
| - Seizures in clusters;  - Rigid muscle tone;  - Cyanosis;  - Deviations of eye and head to one side (variable);  - Bilateral clonic jerks | - Repetitive nocturnal motor seizures in clusters;  -Awakening from sleep;  - Hyperkinetic events with rigid or twisting muscle movements;  - Consciousness maintained during seizures;  - A limited number of seizures during the day | - Recurrent seizures characterized by rigid muscle contractions and rhythmic twitching in the arms and legs;  - Myoclonic spasms;  - Poor quality of sleep;  - Frightening awakenings and episodes similar to nightmares during sleep or at night;  - Uncontrolled movements including sudden movements of the upper limbs during sleep. |
|  |  |  |
| - Onset between 4 and 8 months of age | - Onset in the first two decades of life; mean age ~ 10 years | - Onset on the 2nd day after birth |
| - Seizures typically disappear after 1st year of life | - Lifelong but not progressive | - Patient is 2 years old and still has seizures |
| - Usually normal development;  - Psychomotor arrest is possible in rare cases | - Normal development;  reduced cognitive abilities, intellectual deficits, or coexisting psychiatric conditions may occur | - Developmental delay (speech deficit) |
| - Family history of seizures (similar age at onset, autosomal dominant trait) | - Consistent with autosomal dominant inheritance  ***(note: The absence of a documented family history of ADNFLE does not exclude the possibility of a diagnosis.)*** | - The father, old brother, and old sister with the same mutation variant did not had signs of epilepsy |
