# Supplementary material for: Novel nonsense mutation in gene CHRNA2 identified by whole-genome sequencing in infant with epilepsy disorder: A case report
Source: Heliyon. 2024 Dec 26;11(1):e41484. doi: 10.1016/j.heliyon.2024.e41484 (PMC11743308; doi:10.1016/j.heliyon.2024.e41484)
Supplement: Multimedia component 10 [file mmc10.pdf]

UMC UNIVERSITY Medical Center | НАЦИОНАЛЬНЫЙ НАУЧНЫЙ ЦЕНТР МАТЕРИНСТВА И ДЕТСТВА

Форма ИНФ-32. Версия 1
Утверждена Директором
НЦМД КФ «UMC»
№ 3 от «22» января 2018 г.

ИНФОРМИРОВАННОЕ СОГЛАСИЕ ПАЦИЕНТА НА
проведение молекулярно-генетического исследования

Уважаемый пациент!

В соответствии со статьями 88, 91 Кодекса Республики Казахстан от 18 сентября 2009 года № 193 – IV «О здоровье народа и системе здравоохранения», а также в соответствии с:

Вашим желанием (направлением лечащего врача) у Вас предполагается произвести взятие образца биологического материала (кровь, биопсийный материал и д.р.), который будет использован для молекулярно-генетического исследования (исследования ДНК). Эти исследования помогут выявить носительство некоторых генетических заболеваний или увеличение риска наиболее частых болезней, развитие которых зависит как от наследственных факторов, так и от факторов внешней среды.

Прежде, чем молекулярно-генетическое исследование будет выполнено, Ваш лечащий врач или врач-консультант должны объяснить Вам цель тестирования, его информативность, вероятность получения правильного результата и возможные последствия тестирования (в том числе, возможность практического использования результатов) для Вас или Ваших родственников.

Подписывая данную форму, Вы подтверждаете, что **даете свое согласие** на молекулярно-генетическое исследование добровольно, обладая достаточной информацией, и что у Вас была возможность задать все вопросы, которые могли у Вас возникнуть относительно молекулярно-генетического исследования. Все личные данные, касающиеся Вас или Ваших родственников, являются конфиденциальными, и не могут быть переданы другим лицам иначе, как с Вашего ясно выраженного согласия. Вы можете отозвать свое согласие в любое время без дополнительных объяснений.

Настоящим я выражаю свое согласие на то, что генетическое исследование будет выполнено:

***Мне (моему ребенку, подопечному)** ____________________ ____________
(Фамилия, имя, отчество) *(Дата рождения)*

***Отношение к пациенту** ____мама____ **если пациент в сопровождении*

с целью ____

и биологический материал будет сохранен до получения и оценки результатов, а также для выполнения других дополнительных исследований, если это потребуется для получения четких результатов.

Подпись пациента: ____[подпись]____

Дата заполнения: «____» ______________ 20____года **Время заполнения ____:____**

Врач или сотрудник лаборатории ______________________ **Подпись**__________
(Ф.И.О.)

Дата заполнения: «____» ______________ 20____года **Время заполнения ____:____**

UMC University Medical Center (Science*Education*Healthcare)
Informed Consent of the Patient for Molecular Genetic Testing

Form ИНФ-32. Version 1 Approved by the Medical Director at the UMC Medical Center No. 20 on November 16, 2020

In accordance with Articles 88 and 91 of the Code of the Republic of Kazakhstan dated July 7, 2020, No. 360-VI ЗРК with amendments as of March 31, 2021, "On Public Health and Healthcare System," and also in accordance with:

Your wish (referral from the attending physician), it is proposed to collect a sample of biological material (blood, biopsy material, etc.) from you, which will be used for molecular genetic testing (DNA/RNA analysis). These tests will help identify carrier status of certain genetic diseases or an increased risk of the most common diseases, the development of which depends on both genetic and environmental factors. If the concentration of DNA/RNA is low, it may require a repeat collection of peripheral blood.

Before the molecular genetic test is conducted, your attending physician or consulting physician must explain to you the purpose of the testing, its informativeness, the likelihood of obtaining correct results, and the possible consequences of the testing (including the potential practical use of the results) for you or your relatives.

I agree that the sample may be used for scientific research, provided that it does not lead to the disclosure of personal information about me or my child.

I agree that if necessary, the consulting physician or laboratory staff conducting the research may contact me using the contact information.

By signing this form, you confirm that you consent to the molecular genetic testing voluntarily, having received sufficient information, and that you have had the opportunity to ask any questions that may have arisen regarding molecular genetic testing. All personal data concerning you or your relatives are confidential and cannot be disclosed to other parties except with your explicit consent. You may withdraw your consent at any time without further explanations.
I hereby give my consent for the genetic testing to be performed on:

- Myself (my child, ward) (Last name, first name, patronymic) (Date of birth)
- Relationship to the patient *if the patient is accompanied

For the purpose of storing the biological material until the results are obtained and evaluated, as well as for conducting other additional studies if necessary to obtain clear results.

Patient's Signature: Date of completion: "" *20* Time of completion: __________

Physician or Laboratory Staff Signature (Full Name) Date of completion: "" *20* Time of completion: ___________
